# Drug repurposing in psoriasis, performed by reversal of disease-associated gene expression profiles

**DOI:** 10.1016/j.csbj.2022.10.046

**Published:** 2022-11-09

**Authors:** Faheem Ahmed, Son Gi Ho, Anupama Samantasinghar, Fida Hussain Memon, Chethikkattuveli Salih Abdul Rahim, Afaque Manzoor Soomro, Pratibha, Naina Sunildutt, Kyung Hwan Kim, Kyung Hyun Choi

**Affiliations:** a Department of Mechatronics Engineering, Jeju National University, Republic of Korea; b Department of Electrical Engineering, Sukkur IBA University, Pakistan; c BioSpero, Inc., Jeju, Republic of Korea

**Keywords:** Psoriasis, Drug repurposing, Gene expression and reversal, CMap, Drug perturbagen

## Abstract

Psoriasis is a skin disease which results in scales on the skin caused by flaky patches. Psoriasis is triggered by various conditions such as drug reactions, trauma, and skin infection etc. Globally, there are 125 million people affected by psoriasis and yet there is no effective treatment available, and it emphasizes the need for discovery of efficacious treatments. De-novo drug development takes 10–17 years and $2–$3 billion of investment with <10 % success rate to bring drug from concept to a market ready product. A possible alternative is drug repurposing, which aims at finding other indications of already approved drugs. In this study, a computational drug repurposing framework is developed and applied to differential gene expressions of Psoriasis targets obtained from the publicly available database (GEO). This strategy uses the gene expression signatures of the Psoriasis and compares it with perturbagen available in the CMap. Based on the connected signature drugs are ranked which could possibly reverse the signatures to stop the psoriasis. The drugs with most negative connectivity scores are ranked efficient and vice versa. The top hit drugs are verified using the literature survey of the peer reviewed journal, electronic health records, patents, and hospital database. As a result, 50/150 and 37/150 drugs are confirmed to have anti-psoriasis efficacy in two datasets. Top 10 drugs are suggested as potential repurposable drugs for psoriasis. This study offers, a powerful yet simple approach for rapid identification of potential drug repurposing candidates in Psoriasis and any disease of interest.

## Graphical abstract

**Figure:**
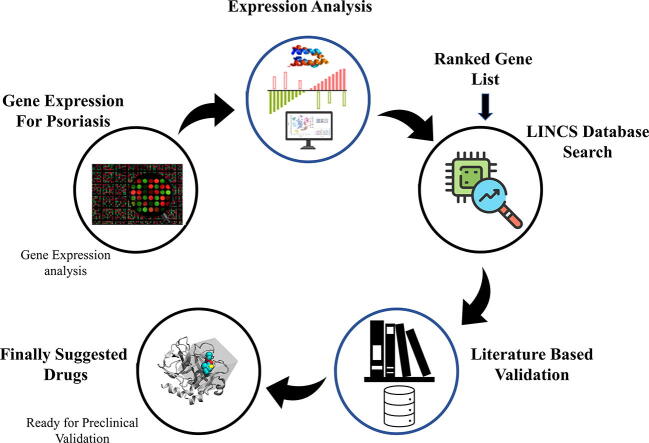

## 1 Introduction

Psoriasis is a common autoimmune disease that results in flaky patches of skin which form scales. It mostly appears on the knees, elbows, and scalp. The autoimmune disorder is linked with hyperproliferation of skin cells and normally known by red itchy spots, thick and scaly skin, and lesions appearing from hyperkeratosis [1]. In psoriatic disease conditions, crosstalk between immune cells and keratinocytes is mediated by the release of cytokines, chemokines, and growth factors. Patients experiencing psoriasis decreases the quality of life (QOL) and ends up in physical or mental imperfection when it is compared to patients suffering from other chronic diseases such as diabetes, depression, heart disease [2]. Moreover, according to data of global epidemiology, patient rate is 2 %–3 % (125 million people worldwide) of the world population [3]. Despite huge number of patients worldwide and huge amount of money being spent on drug research for psoriasis, there is no effective cure for the Psoriasis yet. Despite many efforts, Otezla is the first and only pill approved by FDA back in March 2014 [4]. Otezla is first selective inhibitor of phosphodiesterase 4 (PDE4) indicated for adults with active PsA. It is used to treat severities of plaque psoriasis ranging from mild, moderate, and severe [5].

At this moment, the treatments suggested for psoriasis patients are decided by the severity of the medical condition. To cure the mild psoriatic conditions topical therapies including corticosteroids and vitamin D analogues are used, whereas in worsening disease conditions, combination therapies are practiced. Combination therapies normally comprise of phototherapy along with systemic therapies (methotrexate, cyclosporine, acitretin). More advanced disease needs the oral-modifying antirheumatic drugs (DMARDs) or the TNF-α or 12/23 Interleukin (IL) inhibitors. Despite number of available treatment options, there is no effective treatment to fully eradicate the disease. Traditional way to find the drug/treatment that could eradicate the disease is called de-novo drug development. De-novo drug development takes a new drug molecule and performs all the preclinical validations [6], [7] to confirm the drug safety and efficacy in cell culture and animal models prior to use in human clinical trials. The whole process of de-novo drug development takes 12–17 years with $2∼$3 billion of investment and yet the total success rate is less than 10 % [8], [9]. This renders the de-novo drug development costly, time taking, and extremely risky process. Thus, a feasible alternative to de-novo drug development is drug repurposing which is the process of finding the alternative indications of already approved drugs. It reduces the time by 2–5 years and investment to $200∼$300 million [10], [11], [12].

A lot of work in general has been done in drug repurposing field. Many different techniques have also been used for drug repurposing for psoriasis. Advances in AI and ML have been witnessed for applications in psoriasis and other related conditions [13]. A recent Ensembl machine learning (ML) based study was published to predict the unknow drug-drug interactions (DDIs) for psoriasis [14]. >30 Sources were used for predicting the 37,611 unknown pairs of drugs. Another ML based work where Drug-disease relationships were modelled using the word-embedding of >20 million articles were presented in [15]. ML studies, despite being accurately predicting the DDIs, or drug-disease pairs are limited by lack of expansivity and inability to predict the clinical efficacy. Additionally, docking based study was done in [16] where 2000 FDA approved drugs were docked with 15 anti-psoriatic targets. Twelve drugs were prioritized based on docking score in the range of −12 to −11 kcal/mol. Molecular docking (MD) studies have been widely used in the drug discovery, but MD has some limitations such as incomplete information of binding pockets of the receptors, incomplete geometry, and lack of molecular flexibility etc. [17], [18]. Moreover, studies are also using the psoriasis associated genetic targets from GWAS for drug repurposing such as done in [19]. This study used the GWAS to find the 126 psoriasis related genes and using different tools 68 druggable proteins were identified. Small molecule (Pandal) targeting FDA approved POLI and interleukin 13 (IL 13) [20] were identified as potential therapeutic compound. Similar study is presented in [21] where, a framework was presented to repurpose the drugs for psoriasis using the clinical transcriptomic datasets derived from psoriasis, induced psoriasis-like and drug intervention samples. The molecular mechanisms in psoriasis were explored to find the repurposable compounds. The limitation of this is might the drug be agonist and the target gene are upregulated in medical condition and vice versa. Moreover, it is difficult to distinguish the causal genes in GWAS. To overcome these limitations, a disease-drug transcriptomic reversal approach can be used. Additionally, the previous drug repurposing techniques are using the animal models and in-vitro assays which also have two major limitations. First, these validation tools are sub-optimal representations of human disease and second these methods are time taking and costly [22].

Thus, here a drug-disease transcriptomic reversal-based drug repurposing techniques is proposed by integrating the disease gene signatures from clinical patients obtained from publicly available database (GEO) [23] and drug perturbation data to identify the potentially repurposable compounds. Moreover, the use of control and disease samples of a psoriasis disease signature true state of the disease is captured. The psoriasis signatures are then used to carry out database search in clue.io portal [24] and drug compounds with highest negative values that could possibly reverse the disease. To validate the search results, peer reviewed literature, patents, and clinical database search was carried. Thus, this study represents a robust strategy to enable the psoriasis disease signature-based drug repurposing. Moreover, this method can be deployed to other disease of interest following the same series of steps as done in this study for psoriasis. The proposed mechanism can be generalized to any disease case, and it uses the databases and tools which are open source and easily accessible to smooth the drug repurposing process.

## 2 Materials and methods

In this study, a disease-drug transcriptomic based drug repurposing framework for Psoriasis is presented. The differential gene expression signatures of Psoriasis are downloaded from GEO and for up and downregulated differential gene expression signatures, repurposable drugs that could reversal the expression signatures are found out. Drug expression signatures are taken directly from the LINCs associated clue.io platform. A reversal score based on the Kolmogorov–Smirnov statistic (connectivity score) is generated for each disease-drug pair, with the idea that if the drug profile significantly reverses the psoriasis disease signature, it can stop the disease and be a potential therapy for a psoriasis.

### 2.1 Psoriasis gene expression signatures

**Fig. 1:**
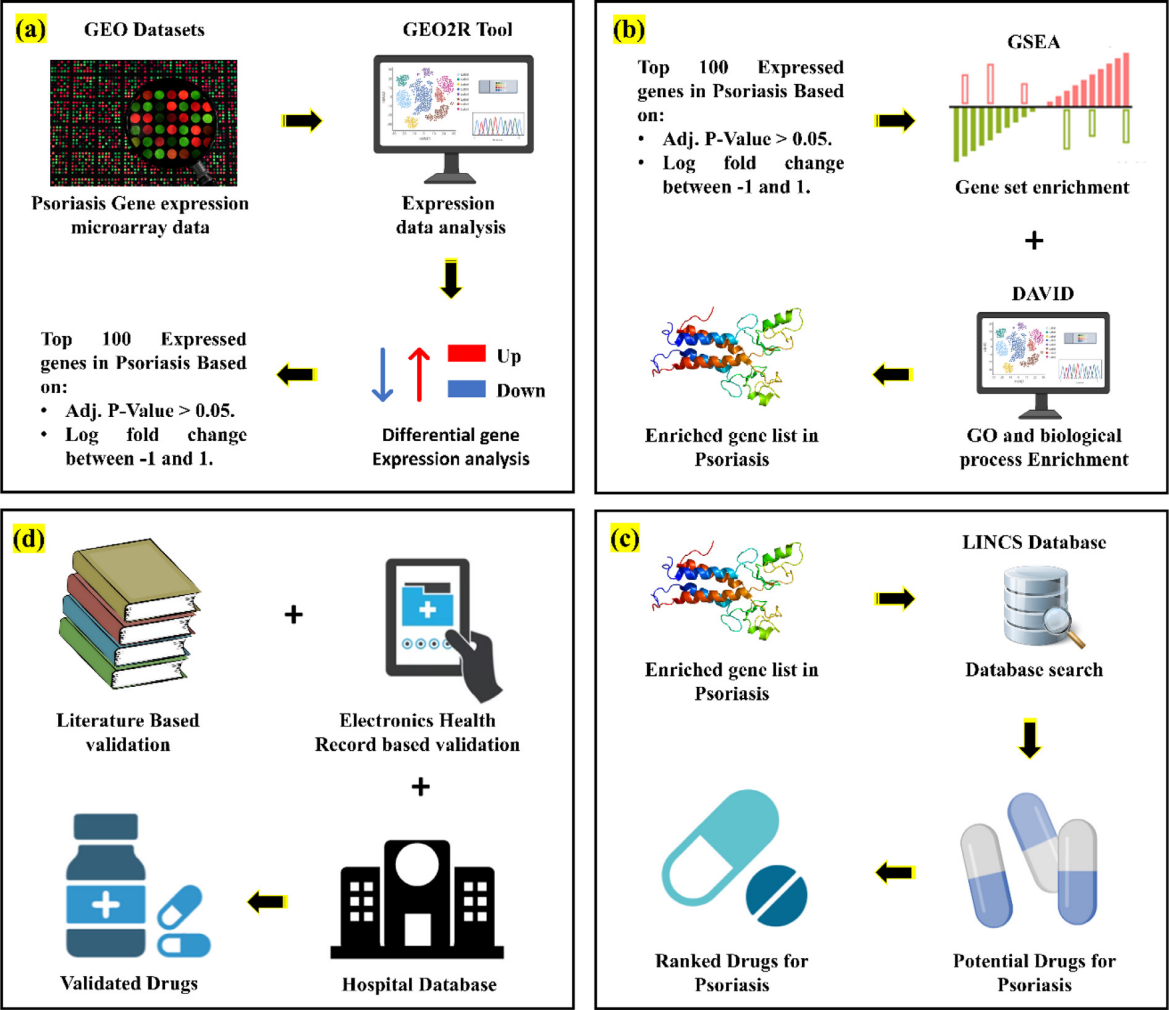
Drug repurposing strategy for Psoriasis. a) Gene expression data obtained from GEO with analysis being performed in GEO2R, b) followed by differential expression analysis and enrichment analysis. c) Database search based on the enriched gene list and d) finally performing validation.

Three different psoriasis related gene expression datasets named GSE13355, GSE14905, and GSE27628 were downloaded from GEO datasets as shown in Fig. 1(a). GSE13355 contains total 180 samples extracted from RNA taken punch biopsies of 58 psoriatic patients and 64 normal healthy controls presented in [25]. All 180 samples were run on Affymetrix HU133 Plus 2.0 microarrays containing >54,000 gene probes. Similarly, GSE14905 consists of a total of 82 samples taken from 54 total subjects. In this dataset skin biopsy samples were collected from 21 normal healthy donors and 28 psoriasis patients with matched lesional and non-lesional tissue presented by the work done in [26] with the purpose of finding the potential therapeutic targets for Psoriasis. Finally, GSE27628 dataset presented by [27] is a result of genome-wide expression profiling of five mouse models. This dataset is comprised of 34 samples in total. 17 samples were presented as control sample and other 17 samples represent disease samples. All the mentioned datasets files are provided as Table S3 and Table S4.

### 2.2 Differential gene expression analysis

All the three datasets were first analyzed using the on-site GEO2R (Fig. 1(a)) tool to perform the differential gene expression (DEG) analysis. Across three datasets, all the normal samples were named as ‘normal’ and disease samples were name as ‘Psoriasis’. Processed files from GEO2R were downloaded for further analysis in Microsoft excel. The adjusted p-value setting of the GEO2R generated files were set below 0.05 while the Log2 fold change range was set between −1 and 1. After the initial analysis DGEs were ranked based on the Log2 fold change followed by the extraction of top 100 upregulated and downregulated genes (Fig. 1(a)). The analyzed DEGs for GSE13355 are mentioned in Table S1, whereas for GSE14905 and GSE27628 top ranked list of DEGs are given in Table S2 and Table S3 respectively. (Adjusted p-value < 0.05, Log2 fold change value between −1 and 1, top ranked genes (Up regulated and down regulated)).

### 2.3 Pathway enrichment analysis

Functional enrichment gene-set analysis was performed using the GSEA [28] software as shown in Fig. 1(b). Genes were pre-ranked using the adjusted p-value and Log2 fold change value. Top 100 genes highly upregulated to downregulated in Psoriasis were fed to GSEA. Moreover, to perform the GSEA the hallmark gene sets (50) were retrieved directly from the MSigDB signature database [28]. This was followed by the KEGG pathway enrichment gene ontology (GO) terms identification of enriched biological process carried out using DAVID online tools (Fig. 1(b)) and database [29]. Further the GO and pathway enrichment was also performed using the Enricher online tool [30].

### 2.4 Clue.io gene expression reversal and scoring

Once the initial data extraction and data analysis is complete, next step is to perform the database search. Since the recent breakthroughs in technology have led to generation of high dimensional perturbational datasets. The huge data integrated across multiple assays, cells, and conditions of dose and treatment time require to be backed by sufficient computational expertise to be addressed. To meet such needs, connectivity map (CMap) [31], [32], [33] has developed a cloud-based software called CLUE for the analysis of perturbational datasets generated using gene expression. Here, CLUE has been used to find the small molecule drugs which could possibly reverse the gene expression signatures. This process was performed by first separating the top upregulated and top downregulated genes in Psoriasis (Fig. 1(a-b)) and the “Query” tool of the clue platform was used to find positive and negative connections between gene expression signature of psoriasis genes and all the signatures in CMap to find the potential repurposable compounds (Fig. 1(c)). The drug scoring algorithm used by CMap LINCS gene expression resources is referred to as connectivity score. It is a value ranging from −100 to +100 and it elucidates the connection between the disease associated queried signature and a drug perturbagen. Connectivity score usually contains three components: 1) False discovery rate (FDR) followed by 2) a nominal p-value, and 3) τ, which expresses the effect size of a mentioned enrichment score. τ is a dimensionless measure ranging from −100 to +100. Moreover, FDR adjust the p-value to use it for various hypothesis validation when the large number of comparisons are given. Similarly, p-value depicts the significance and similarity of the reference versus null distribution of the random queries. Next, the drugs were ranked, and top scoring drugs were taken for downstream analysis as shown in Fig. 1(c).

### 2.5 Statistical analysis

**Fig. 2:**
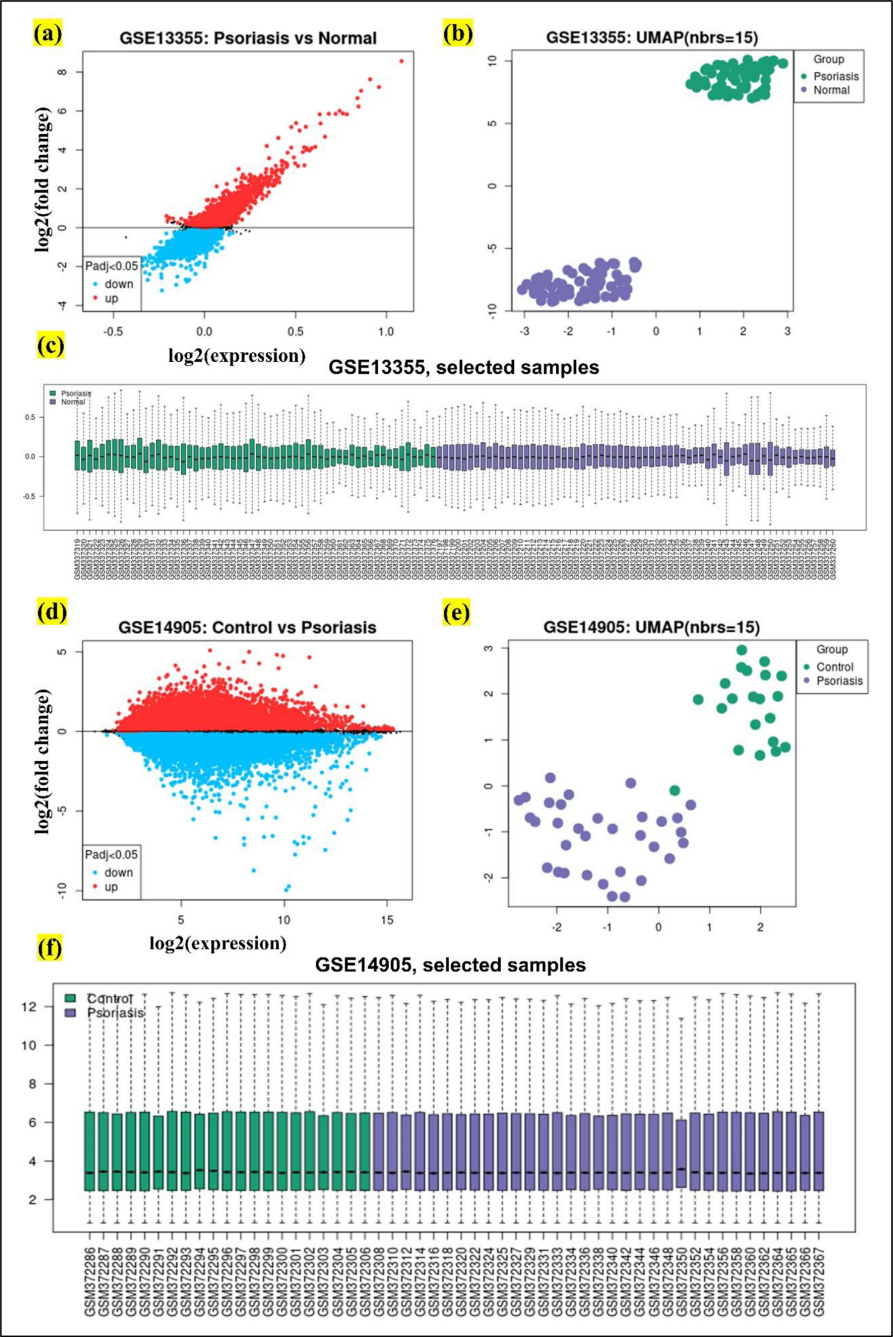
GEO2R results for Psoriasis gene expressions. a) shows the psoriasis genes in normal vs psoriasis patients, b) shows the cluster of the Psoriasis vs normal individuals in the dataset with identifier GSE13355, and c) depicts the total number of selected healthy vs disease samples. Similarly, d) shows the psoriasis genes in normal vs psoriasis patients for GSE14905, e) clustering results of the total number of healthy vs psoriasis patients in the dataset, and finally f) presents the total number of control and psoriasis patient samples.

For initial analysis of the gene expression profiles, GEO2R, a R based tool was used and to find out the differential gene expression adjusted p-value and Log2 fold change (Fig. 2) values were used [34]. Two different, psoriasis associated datasets identified with GSE13355 and GSE14905 were downloaded from the GEO database. Fig. 2 shows the complete results obtained from the GEO2R tool. These results show the gene expression in psoriasis vs normal subjects as shown in Fig. 2 (a & d). Similarly, clustering is also performed individually for both gene expression datasets with GEO identifiers as GSE13355 and GSE14905. Moreover, the gene expression datasets were clustered by GEO2R tool based on the psoriasis vs normal gene expression data and the resulting density of the expression in diseased vs normal samples are shown in Fig. 2 (b & e). Additionally, the results of positive and negative data samples obtained from both gene expression datasets are given in Fig. 2 (c & f). Later the genes, with Log2 fold change values in positive range were rendered as upregulated in Psoriasis while with negative values were called downregulated in Microsoft excel while setting the p value range to <0.05 and log2 fold change range of −1 and 1. Whereas the drugs because of query in “Clue” were ranked using the reversal scores. The more negative result for a drug, means the drug has higher potential to reverse the expression profiles. With this connections, top drugs with highest negative values were shortlisted for further analysis as shown in Fig. 1(c).

### 2.6 Validation of the ranked drugs

To validate the ranked drugs, literature search was done. The peer reviewed journals, databases, and patents were mainly focused [11], [35], [36]. To avoid the bias, evidence was extended to positive and negative results for a given potential drug. To further elaborate, to handle the biases, search was carried out in two steps: i) Sources supporting the use of drug(s) for psoriasis and ii) Sources denying the use of drug(s) for psoriasis. According to the mentioned steps, if for any drug step (i) was true then it was termed as validated, if both steps were true then it was termed as not confirmed and were eliminated from further consideration, and finally if both steps were false for any drug, it was termed as potential candidate to be preclinically and clinically validated. In this the chances of false-positive or false-negative were minimized. Additionally, electronic health records and hospital databases were also traversed to find if the ranked drugs have already been used in psoriasis or not.

## 3 Results and discussions

In this study, the developed repurposing pipeline was applied to psoriasis gene expression profiles to find the repurposable drugs that could potentially reverse the gene expressions and finally lead to inhibition of disease as shown in Fig. 1. The repurposed drugs were later validated through literature and databases to confirm if the predicted potential drugs have already been used for the psoriasis or not.

### 3.1 Using gene expressions to find drug repurposing candidates

To compute and find the disease associated gene-expression data we queried the public database (GEO) with disease-related terms. The resulting expressions that were shortlisted included the disease-associated gene expression changes [37], [38]. gene expression datasets named GSE13355, GSE14905, and GSE27628 were downloaded from GEO and after performing p-test using GEO2R, top 100 genes both upregulated and downregulated were selected with adjusted p-value of < 0.05 and Log2FC value of −1 to 1. The analyzed DEGs for GSE13355 are mentioned in Table S3 and for GSE14905 top ranked list of DEGs are given in Table S4.

Later these gene expressions were used to perform the database search in CMap database to find the compounds that could reverse these gene expression profiles. Top 150 repurposable drugs with highest potential to reverse the gene expression profiles were analyzed for GSE13355 (Table S1) and for GSE14905 (Table S2). Here, drugs with lowest score (−99) are referred to as high potential drugs that are expected to reverse the gene expression profiles and drugs with highest positive score (99) are referred to as the drugs which could promote the expression of the genes.

### 3.2 Gene set enrichment analysis

**Fig. 3:**
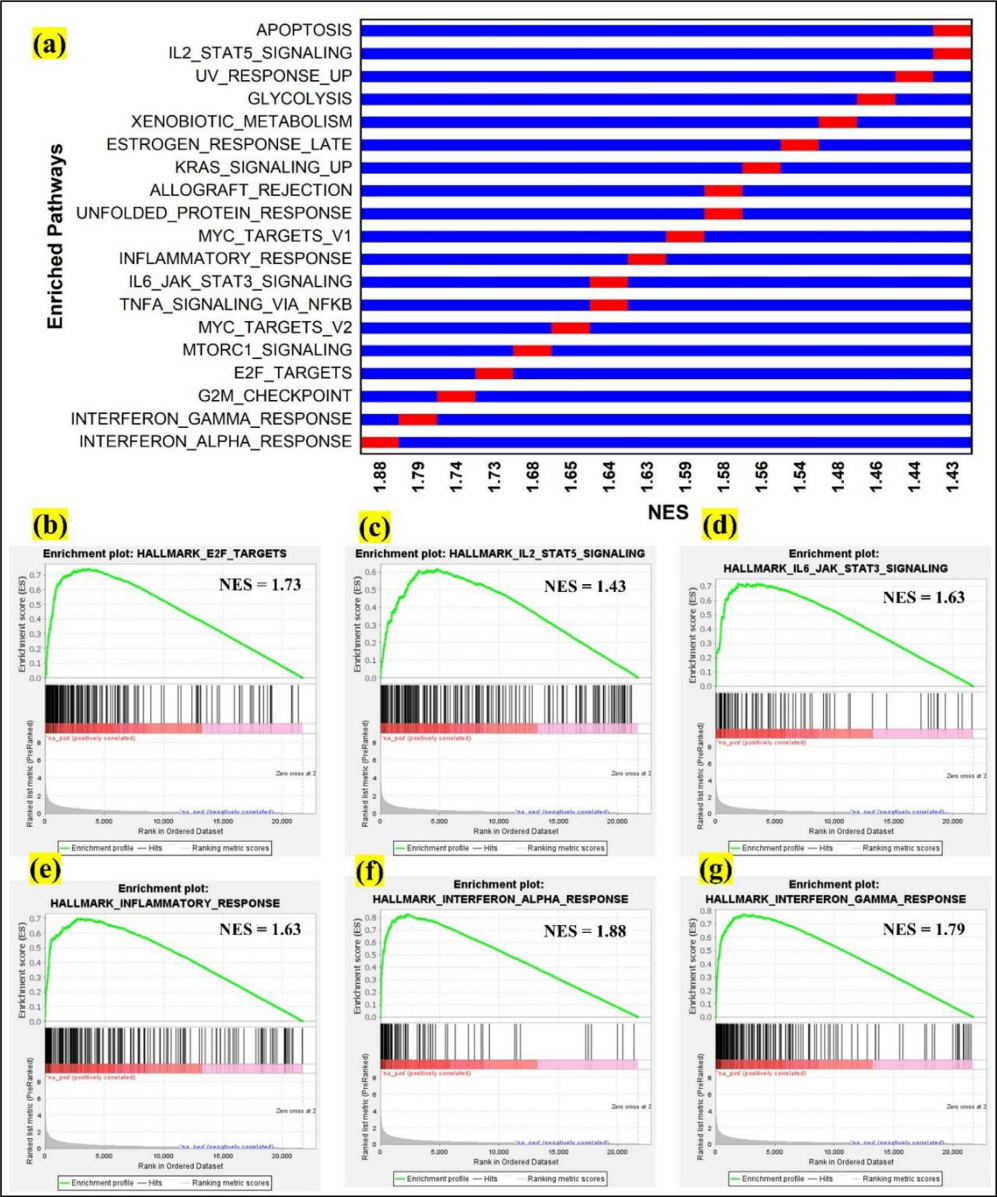
Gene set enrichment analysis. a) enrichment of pathways which shows the upregulated and downregulated pathways in psoriasis, b-g) the upregulated pathways including E2F, STAT5, STAT3, Inflammatory response, Interferon alpha, and Interferon gamma and their net enrichment scores respectively.

We used GSEA (Gene set enrichment analysis) to perform the annotation of enriched hallmark pathways from each input signature as shown in Fig. 3 (a). This was performed for both datasets (GSE13355 and GSE14905) and E2F, STAT5, STAT3, Interferon alpha, Interferon gamma, and inflammatory pathways were found upregulated (Fig. 3 (b-g)). MTORC1 signaling, oxidative phosphorylation, P53, and spermatogenesis pathways were found downregulated. For the upregulated pathways, enrichment score is positive (Fig. 3 (b-g)) and for the downregulated pathways the score is negative.

### 3.3 Literature based validation

**Fig. 4:**
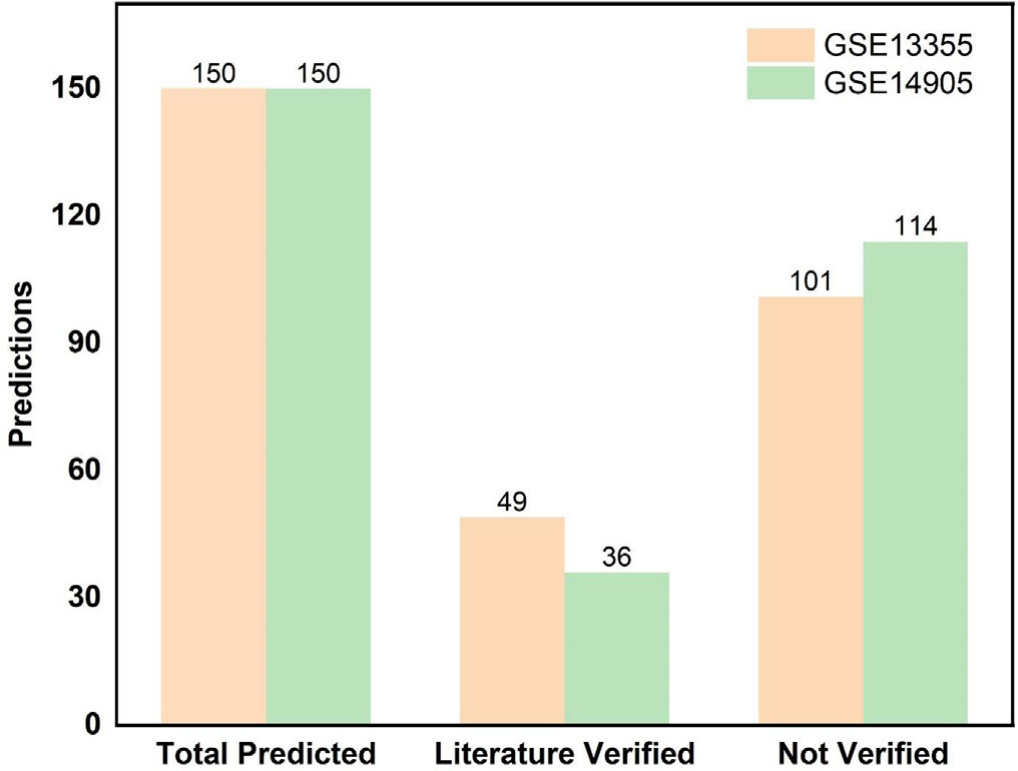
Depiction of number of predicted drugs followed by literature verified and not-verified drugs from both psoriasis expression datasets.

**Fig. 5:**
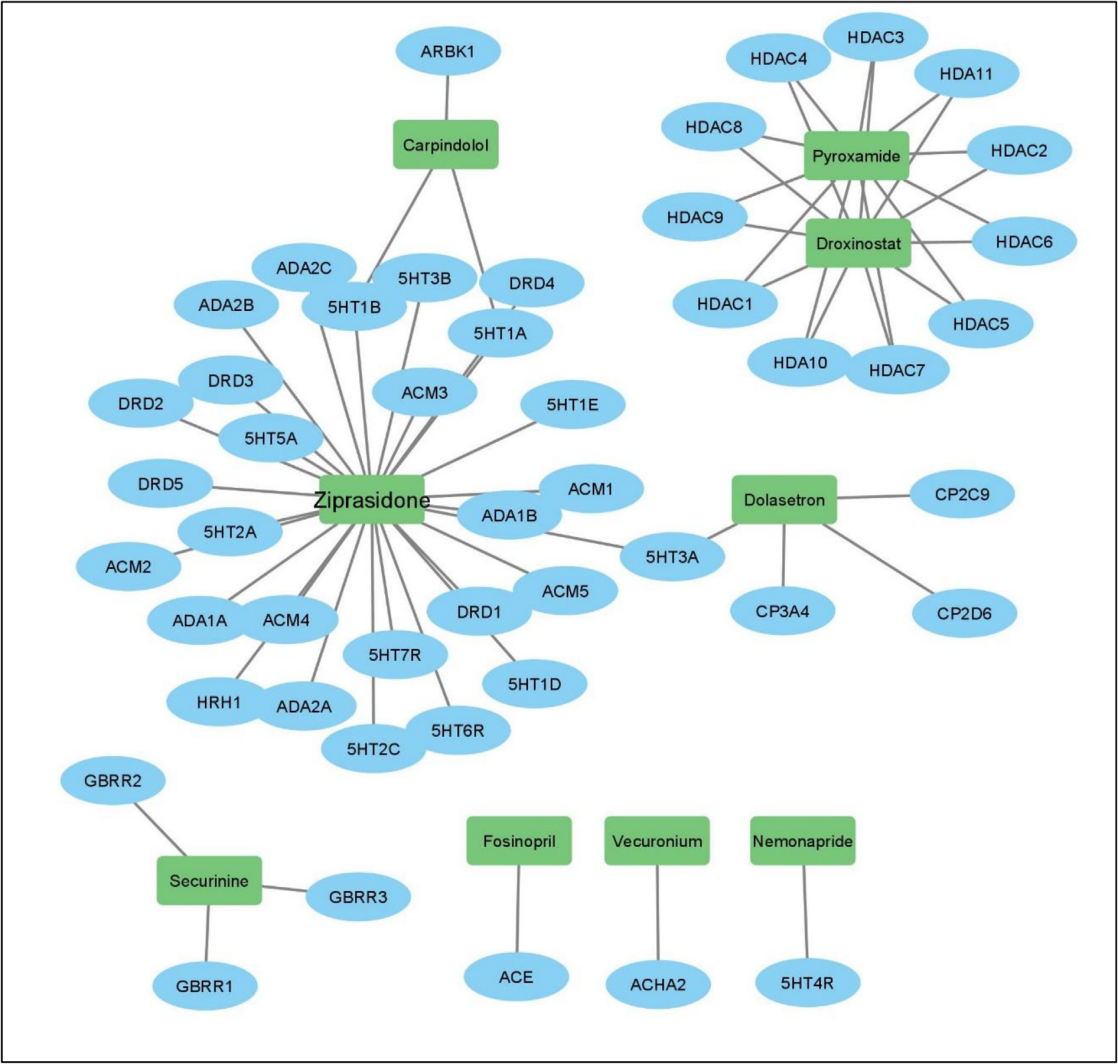
A network of drug-protein targets of the potential drugs repurposable for psoriasis. The drug targets used in the workflow are obtained from the DrugBank database. Moreover, the nodes in the green color represent the drugs whereas the nodes highlighted in the sky blue color represent the drug target proteins. (For interpretation of the references to color in this figure legend, the reader is referred to the web version of this article.)

Literature search for Top 150 repurposable drugs from GSE13355 (Table 1) and GSE14905 (Table 2) was carried out in peer-reviewed journals and databases. The target was to find the any research study that have already use these drugs for psoriasis in pre-clinical or clinical setting. In case the drug is used, it is considered as a true hit and if there is no evidence for a drug in literature or database it means that it could be checked in pre-clinically (in-vitro and in-vivo) [39], [40] to check if the drug could work against psoriasis. As a result of this validation, it was found that 50/150 drugs from GSE13355 and 37/150 for GSE14905 have already been used for the psoriasis as shown in Fig. 4. This confirms that, the approach used is potential and can correctly identify the drugs repurposable for psoriasis [41]. Among unvalidated drugs, top 10 drugs based on the criteria (Known indication and top score) are suggested as repurposable drug candidate for psoriasis (Table 3 and Fig. 5). Fig. 5 shows the drug-target network of the suggested potential drugs repurposable for psoriasis and the network is created in the Cytoscape software after downloading the targets of the drugs from DrugBank. The target information of these drugs is obtained from DrugBank database. At the same time, the approach mentioned here could be extended to other disease of interest as well. Moreover, for the drugs that could not be confirmed from the literature survey, these should be first pre-clinically validated prior to use in humans. Finally, the limitations of the repurposing strategy are acknowledged such as missing values in gene expression profiles, uncertain conditions involved during carrying out experiments, and involved human error. Additionally, the mentioned limitations would be carried out as potential future work.

**Table 1:** Literature based validated drugs shown to have anti-psoriasis activity. (GSE13355).

	**Drug Name**	**Description**	**Original indication**	**Literature based validation**	**Score**
1	Tacedinaline	HDAC inhibitor	Lung Cancer	Acne Vulgaris	−93.05
2	GSK-3-inhibitor-II	PKC inhibitor	Neurodegenerative and psychiatric disorders	Psoriasis	−91.79
3	Cefpodoxime	Bacterial cell wall synthesis inhibitor	Various bacterial infections	Skin and soft tissue infections	−86.11
4	Alimemazine	Histamine receptor agonist	Allergic conditions	Dermatology	−83.47
5	Phylloquinone	Vitamin K	Psoriasis	Psoriasis	−79.42
6	Masitinib	KIT inhibitor	Tyrosine-kinase inhibitor	Psoriasis	−78.03
7	Dihydrexidine	Dopamine receptor agonist	Treatment of SPD – Trial	Psoriasis	−73.76
8	Sertraline	Serotonin receptor antagonist	Major depressive disorder	Psoriasis	−69.52
9	Domperidone	Dopamine receptor antagonist	Agent for dyspepsia, indigestion, epigastric pain, nausea, and vomiting	Psoriasis	−67.74
10	Norcyclobenzaprine	Adrenergic receptor agonist	Musculoskeletal conditions	Dermatology	−66.9
11	Sunitinib	FLT3 inhibitor	Advanced renal cell carcinoma (RCC)	Psoriasis	−66.25
12	Berbamine	Calmodulin antagonist	Various parasitic and fungal infections	Psoriasis	−65.16
13	Reserpine	Vesicular monoamine transporter inhibitor	Treatment of hypertension	Psoriasis	−63.34
14	Rimcazole	Sigma receptor antagonist	Antipsychotic	Psoriasis	−63.26
15	Ivermectin	GABA receptor agonist	Anti-parasite medication	Psoriasis	−61.68
16	Ramipril	ACE inhibitor	Mild to severe hypertension	Psoriasis	−60.09
17	Zalcitabine	Nucleoside reverse transcriptase inhibitor	Used to treat HIV	Psoriasis	−58.5
18	Serdemetan	MDM inhibitor	Treatment of Neoplasms – Trial	Psoriasis	−57.19
19	Gefitinib	EGFR inhibitor	Metastatic non-small cell lung cancer	Psoriasis	−56.89
20	Scriptaid	HDAC inhibitor	Enhance response of human tumor cells to radiation	Psoriasis	−55.84
21	Amlodipine	Calcium channel blocker	Hypertension and angina	Psoriasis	−55.04
22	Fluvoxamine	Selective serotonin reuptake inhibitor (SSRI)	Obsessive Compulsive Disorder (OCD)	Psoriasis	−55.02
23	Epothilone	Microtubule inhibitor	Treatment in various cancers	Psoriasis	−51.7
24	Triptolide	RNA polymerase inhibitor	HIV, Crohn's Disease, Intestinal Diseases - Trial	Psoriasis	−51.55
25	Hispidin	PKC inhibitor	Rheumatoid arthritis	Psoriasis	−49.69
26	Cyclosporin-a	Calcineurin inhibitor	Organ rejection in organ transplant	Psoriasis	−48.59
27	Ritonavir	HIV protease inhibitor	HIV infection	Psoriasis	−47.72
28	Mepacrine	Cytokine production inhibitor	Giardiasis and cutaneous leishmaniasis	Psoriasis	−46.06
29	Brimonidine	Adrenergic receptor agonist	Lowering intraocular pressure	Dermatological	−45.88
30	Norethisterone	Progesterone receptor agonist	Contraceptive when given as monotherapy	Psoriasis	−45.71
31	Pinocembrin	CYP1B1 inhibitor	–	Psoriatic dermatitis	−45.62
32	Carprofen	Cyclooxygenase inhibitor	Pain reliever in the treatment of joint pain and post-surgical pain	Psoriatic-Like Dermatitis	−45.6
33	Seneciphylline	Cytochrome P450 inhibitor	Component of Chinese medicine herb	Psoriasis	−44.78
34	Nelfinavir	HIV protease inhibitor	HIV infection	Psoriasis	−44.64
35	Acepromazine	Dopamine receptor antagonist	Antipsychotic agent	Psoriasis	−43.03
36	Bifonazole	Sterol demethylase inhibitor	Various topical fungal infections	Psoriasis	−42.7
37	Pramipexole	Dopamine receptor agonist	Parkinson’s disease	Psoriasis	−42.49
38	Rolitetracycline	Bacterial 30S ribosomal subunit inhibitor	Broad-spectrum tetracycline antibiotic	Psoriasis	−42.2
39	Flucloxacillin	Bacterial cell wall synthesis inhibitor	Bacterial infection by susceptible microorganisms	Psoriasis	−41.16
40	Ganciclovir	DNA polymerase inhibitor	Cytomegalovirus	Psoriasis	−40.02
41	Bupropion	Dopamine uptake inhibitor	Major depressive disorder (MDD)	Psoriasis	−39.97
42	Fraxidin	Carbonic anhydrase inhibitor	Antibacterial activity against Bacillus subtilis	Psoriasis	−39.27
43	Piperacillin	Bacterial cell wall synthesis inhibitor	Polymicrobial infections	Psoriasis	−38.49
44	Bis-tyrphostin	EGFR inhibitor	–	Psoriasis	−38.4
45	Ribavirin	Antiviral	Treatment of chronic Hepatitis C virus (HCV)	Psoriasis	−37.55
46	Sunitinib	PLK inhibitor	Renal cell carcinoma (RCC)	Psoriasis	−37.49
47	Testosterone	Androgen receptor agonist	Primary hypogonadism and hypogonadotropic hypogonadism	Psoriasis	−37.05
48	Atorvastatin	HMGCR inhibitor	Treatment of several types of dyslipidemias	Psoriasis	−36.48
49	Prostaglandin-e1	Prostanoid receptor agonist	–	Psoriasis	−36.42

**Table 2:** Literature based validated drugs shown to have anti-psoriasis activity. (GSE14905).

	**Drug name**	**Description**	**Original indication**	**Literature based validation**	**Score**
1	Parthenolide	Nfkb pathway inhibitor	Diagnostic of allergic contact dermatitis	Psoriasis	−97.18
2	Voriconazole	Cytochrome P450 inhibitor	Esophageal candidiasis, cadidemia	Psoriasis	−94.7
3	Diazepam	Benzodiazepine receptor agonist	Anxiety, seizures, alcohol withdrawal syndrome	Psoriasis	−93.82
4	Androstenedione	Cytochrome p450 inhibitor	Dietary supplement	Psoriasis	−89.91
5	Levetiracetam	Calcium channel blocker	Seizures in epileptic patients	Psoriasis	−89.15
6	Enzastaurin	Pkc inhibitor	Brain cancer, lymphoma (non-hodgkin's), and lung cancer.	Psoriasis	−88.53
7	Glimepiride	Insulin secretagogue	Type 2 diabetes	Psoriasis	−87.86
8	Quetiapine	Dopamine receptor antagonist	Schizophrenia and depression	Psoriasis	−87.63
9	Isoliquiritigenin	Guanylate cyclase activator	–	Psoriasis	−87.47
10	Pepstatin	Aspartic protease inhibitor	lung diseases, including hypersensitivity pneumonitis	Psoriasis	−86.82
11	Etomoxir	Carnitine palmitoyltransferase inhibitor	Inhibits complex i of the electron transport chain	Psoriasis	−85.3
12	Alfacalcidol	Vitamin d receptor agonist	Hypocalcemia, secondary hyperparathyroidism, and osteodystrophy	Psoriasis	−82.17
13	Benzylpenicillin	Penicillin binding protein inhibitor	Septicemia, meningitis, pericarditis,	Psoriasis	−78.79
14	Chelidonine	Tubulin inhibitor	–	Psoriasis	−77.06
15	Perindopril	ACE inhibitor	Hypertension, mild to moderate congestive heart failure	Psoriasis	−76.6
16	Liothyronine	Thyroid hormone stimulant	Hypothyroidism, nontoxic goiter, myxedema and myxedema coma	Psoriasis	−72.64
17	Tofacitinib	Jak inhibitor	Rheumatoid arthritis (ra), active psoriatic arthritis	Psoriasis	−72.22
18	Liquiritigenin	Aromatase inhibitor	–	Psoriasis	−69.64
19	Niacin	Nad precursor with lipid lowering effects	To reduce elevated tc, ldl-c, apo b and tg levels, and to increase hdl-c in patients with primary hyperlipidemia and mixed dyslipidemia	Psoriasis	−66.06
20	Taurodeoxycholic-acid	Bile acid	Cholestasis	Psoriasis	−64.91
21	Metanephrine	Epinephrine metabolite	Indicated in the emergency treatment of type i allergic reactions	Psoriasis	−64.83
22	Taurocholic-acid	Bile acid	–	Skin inflammation	−63.99
23	Retinol	Retinoid receptor ligand	Vitamin a deficiency	Not good for psoriasis	−62.7
24	Meloxicam	Cyclooxygenase inhibitor	Osteoarthritis, rheumatoid arthritis and moderate to severe pain	Inflammatory conditions	−62.14
25	Ganglioside	Src activator	Modulation of membrane proteins and ion channels, in cell signaling and in the communication among cells	Proliferation of cultured keratinocytes	−60.73
26	Dienestrol	Estrogen receptor agonist	Atrophic vaginitis and kraurosis vulvae	Psoriasis, arthritis	−58.84
27	Hypericin	Tyrosine kinase inhibitor	Inhibit the action of the enzyme dopamine β-hydroxylase, leading to increased dopamine levels	Psoriasis and cutaneous *t*-cell lymphoma	−56.14
28	Leu-enkephalin	Opioid receptor agonist	–	Lesional psoriasis	−56.05
29	Solanine	Acetylcholinesterase inhibitor	Fungicidal and pesticidal properties	Psoriasis and inflammatory conditions	−54.01
30	Pyrrolidine-dithiocarbamate	Nfkb pathway inhibitor	Chelation, induction of g1 phase cell cycle arrest	Psoriasis	−53.35
31	Phenolphthalein	Indicator dye	–	Dermatology	−52.75
32	Vinorelbine	Tubulin inhibitor	Non-small cell lung cancer (nsclc)	Psoriatic skin lesions	−52.3
33	Ofloxacin	Bacterial dna gyrase inhibitor	Treatment of infections (respiratory tract, kidney, skin, soft tissue, uti), urethral and cervical gonorrhoea.	Infections of the skin	−51.38
34	Hydroquinine	Antiarrhythmic	–	Dark skin patches, and psoriasis	−49.53
35	Prostaglandin	Prostanoid receptor antagonist	Platelet-activating-factor-inhibitor	Psoriatic lesions	−48.42
36	Phenamil	Trpv antagonist	Modulator of adipocyte differentiation and pparγ expression	Psoriasis	−46.02

**Table 3:** Top drugs repurposable for psoriasis.

	**Drug name**	**Description**	**Original Indications**	**Repurposable drug indication**	**Score**
GSE13355					
1	Pyroxamide	HDAC inhibitor	Treatment of Leukemia	Psoriasis	−92.31
2	Droxinostat	HDAC inhibitor	Histone Deacetylase Inhibitor	Psoriasis	−91.57
3	Dolasetron	Serotonin receptor antagonist	Used in chemotherapy and postoperatively.	Psoriasis	−89.02
4	Ecopipam	Dopamine receptor antagonist	Treatment of Tourette's Syndrome	Psoriasis	−85.97
5	Nemonapride	Dopamine receptor antagonist	Treatment in gastroesophageal reflux disease	Psoriasis	−77.88

GSE14905					
6	Vecuronium	Acetylcholine receptor antagonist	Muscle relaxing agent and is used as an adjunct in general anesthesia.	Psoriasis	−93.31
7	Fosinopril	ACE inhibitor	Hypertension, congestive heart failure, slower the rate of progression of renal disease	Psoriasis	−90.19
8	Ziprasidone	Dopamine receptor antagonist	Schizophrenia	Psoriasis	−89.38
9	Securinine	GABA receptor antagonist	Neurological disorder	Psoriasis	−89.01
10	Carpindolol	Adrenergic receptor antagonist	Heart failure with reduced ejection fraction	Psoriasis	−88.58

## 4 Conclusion

In this study, a drug repurposing pipeline is presented, that uses the gene expression profiles of disease (psoriasis) to find the potential repurposable drugs for psoriasis by performing database search in cMAP. The gene expression profiles (downloaded from GEO) used here were obtained from human lesional and non-lesional skin or from the psoriatic patients and normal human controls. After performing the initial analysis (p < 0.05 and Log2FC = -1 to 1), top 100 up and down regulated genes were taken to query the database. It was performed individually for all the datasets. The resulting drugs were ranked using the gene reversal score (connectivity score) and top 50 hits (Score −99 or higher) for each dataset were download. Literature survey indicated that 50/150 drugs from GSE13355 and 37/150 for GSE14905 has already been validated by the researchers in pre-clinical and clinical settings. Among unvalidated drugs, top 10 drugs based on the criteria (Known indication and top score) are suggested as repurposable drug candidate for psoriasis (Table 3). For the drugs that were not validated through literature, it is highly recommended to first validate them in related pre-clinical assays (in-vitro) prior to use them in humans. Despite successful results, the limitations of the work are acknowledged which can be taken as potential future directions. These limitations include the chances of biasness in literature-based confirmation, variability of conditions in which the gene expression data in experimental and clinical setting is obtained, and the basic hypothesis of the study is that the reversal of gene expression profiles will reverse the disease which is not the case sometimes. Additionally, as a potential future work, the drug repurposing framework will be done fully automated so that the activity speed can be increased, and larger community can benefit from. Despite these limitations, the presented approach yielded the successful results and if similarly followed, it can be applied to any disease of interest. This implies that, drug repurposing workflow proposed in this study can be generalized to any disease of interest by finding the disease associated gene expression data first followed by the analysis of the gene expression, gene set enrichment analysis (GSEA), and LINCS database search. Finally, the results would be prioritized based on connectivity score and after literature search potential repurposable drugs would be recommended for preclinical and clinical validation prior to use in humans.

## CRediT authorship contribution statement

**Faheem Ahmed:** Conceptualization, Formal analysis, Writing – review & editing. **Son Gi Ho:** Conceptualization, Formal analysis, Writing – review & editing. **Anupama Samantasinghar:** Conceptualization, Writing – review & editing. **Fida Hussain Memon:** Formal analysis, Investigation, Validation. **Chethikkattuveli Salih Abdul Rahim:** Validation. **Afaque Manzoor Soomro:** Formal analysis, Investigation, Validation. **Pratibha:** Validation. **Naina Sunildutt:** Writing – review & editing. **Kyung Hwan Kim:** Writing – review & editing. **Kyung Hyun Choi:** Conceptualization, Resources, Supervision, Funding acquisition.

## Declaration of Competing Interest

The authors declare that they have no known competing financial interests or personal relationships that could have appeared to influence the work reported in this paper.
